# Mapping the EORTC QLQ-C30 to EQ-5D-3L in patients with breast cancer

**DOI:** 10.1186/s12885-021-08964-5

**Published:** 2021-11-18

**Authors:** Laura A. Gray, Monica Hernandez Alava, Allan J. Wailoo

**Affiliations:** Health Economics and Decision Science, School of Health and Related Research, University of Sheffield, Sheffield, UK

**Keywords:** QLQ-C30, EQ-5D-3L, Utility mapping, Mixture models, ALDVMM

## Abstract

**Background:**

The types of outcomes measured collected in clinical studies and those required for cost-effectiveness analysis often differ. Decision makers routinely use quality adjusted life years (QALYs) to compare the benefits and costs of treatments across different diseases and treatments using a common metric. QALYs can be calculated using preference-based measures (PBMs) such as EQ-5D-3L, but clinical studies often focus on objective clinician or laboratory measured outcomes and non-preference-based patient outcomes, such as QLQ-C30. We model the relationship between the generic, preference-based EQ-5D-3L and the cancer specific quality of life questionnaire, QLQ-C30 in patients with breast cancer. This will result in a mapping that allows users to convert QLQ-C30 scores into EQ-5D-3L scores for the purposes of cost-effectiveness analysis or economic evaluation.

**Methods:**

We use data from a randomized trial of 602 patients with HER2-positive advanced breast cancer provided 3766 EQ-5D-3L observations. Direct mapping using adjusted, limited dependent variable mixture models (ALDVMM) is compared to a random effects linear regression and indirect mapping using seemingly unrelated ordered probit models. EQ-5D-3L was estimated as a function of the summary scales of the QLQ-C30 and other patient characteristics.

**Results:**

A four component mixture model outperformed other models in terms of summary fit statistics. A close fit to the observed data was observed across the range of disease severity. Simulated data from the model closely aligned to the original data and showed that mapping did not significantly underestimate uncertainty. In the simulated data, 22.15% were equal to 1 compared to 21.93% in the original data. Variance was 0.0628 in the simulated data versus 0.0693 in the original data. The preferred mapping is provided in Excel and Stata files for the ease of users.

**Conclusion:**

A four component adjusted mixture model provides reliable, non-biased estimates of EQ-5D-3L from the QLQ-C30, to link clinical studies to economic evaluation of health technologies for breast cancer. This work adds to a growing body of literature demonstrating the appropriateness of mixture model based approaches in mapping.

**Supplementary Information:**

The online version contains supplementary material available at 10.1186/s12885-021-08964-5.

## Background

There is often a disparity between the requirements of cost-effectiveness analysis and evidence of their efficacy from clinical studies. One area where this occurs relates to the types of outcomes measured compared to those required for decision making. In many jurisdictions, decision makers routinely use quality adjusted life years (QALYs) to compare the benefits and costs of treatments across different diseases and different treatments using a common metric. A year spent in full health is represented with a value of 1 QALY. QALYs can be calculated using preference-based measures (PBMs) of health outcomes, or health utilities, but clinical studies often focus on objective clinician or laboratory measured outcomes and non-preference-based patient outcomes.

This type of evidence gap is particularly apparent in breast cancer where studies of new pharmaceuticals often focus on time to disease progression, survival and cancer specific measures of quality of life. Preference-based measures are administered and reported much less frequently. In this situation, one solution that analysts can employ to enable a cost per QALY analysis to be undertaken is to estimate preference-based health utility values that would have been observed had such measures been administered, from the outcomes that were. This linking of clinical and economic outcome measures requires data from outside the clinical studies of treatment efficacy and is typically referred to as mapping. It is a technique in widespread use, underpinning many economic evaluations in different disease areas, including breast cancer [1–5]. In evaluations of health technologies for patients with breast cancer mapping has previously been used, for example in economic models considered as part of the NICE technology appraisal process [6] and has been widely used to bridge this evidence gap in other disease areas.

Often, very simple linear models have been used to estimate these PBMs from clinical measures. However, linear models have been shown to underperform and fit data poorly in this type of analysis and when used to estimate PBMs which have non-standard distributions due to their unusual scale [5, 7–9]. Generally these models overestimate PBMs in those with poor health and underestimate it in those with better health. This could mean that the benefits to patients of new treatments for breast cancer are undervalued and appear less cost-effective, often meaning that patients have less access to these treatments.

In this paper, we map from the EORTC QLQ-C30 to EQ-5D-3L, UK tariff, using bespoke mixture models and compare results to other mapping methods in a sample of breast cancer patients. Using data from the TH3RESA breast cancer trial, we compare this technique with response mapping which has also been shown to outperform many statistical techniques previously used in utility mapping [10], as well as to a linear model. EQ-5D-3L is the most commonly used PBM used to measure generic health utilities. It comprises five dimensions (mobility, self-care, usual activities, pain, anxiety/depression) with three levels for each dimension (no problems, moderate problems, extreme problems). The European Organisation for Research and Treatment of Cancer (EORTC) quality of life questionnaire (QLQ) assesses health-related quality of life (QoL) of patients with cancer participating in international clinical trials. The QLQ-C30 core questionnaire is commonly used in clinical trials and other clinical studies and is made up of 30 questions [11]. It includes single-item measures as well as multi-item scales; these include a global health status (QoL) scale, five functional scales for physical, role, emotional, cognitive and social functioning, three symptom scales for symptoms of fatigue, nausea and vomiting and pain, and six single-item measures for dyspnoea, insomnia, appetite, constipation, diarrhoea, and financial difficulties. Each of these are conveyed on a 0–100 scale where higher levels of functioning, QoL or levels of symptoms are represented by a higher score.

There are studies which have mapped QLQ-C30 to EQ-5D in patient with breast cancer previously. Crott and Briggs performed mapping from the QLQ-C30 version 1.0 to the UK EQ-5D tariff using approximately 800 observations from patients with locally advanced breast cancer in a randomized controlled trial [3]. Kim et al. also used OLS in a study using data from 149 patients with breast cancer, and mapped QLQ-C30 to EQ-5D-3L [12]. However, the results in each of these mapping studies exhibited some degree of bias. All used linear regression methods that have been shown repeatedly in other applications to underestimate health utility for those in good health and overestimate it for those in poor health [7, 13]. The distribution of EQ-5D has a number of non-standard characteristics. It is multi-modal, has a large spike at 1 (representing full heath) and a gap between full health and the next feasible value. It also has a lower bound at − 0.594. Other studies have mapped QLQ-C30 to EQ-5D in cancer patients, some of whom had breast cancer and compared mapping using OLS to other regression techniques including 2-part models, Tobit models, response mapping and polynomial splines [1, 14, 15]. However, all of the techniques compared in these papers have failed to address all of these characteristics simultaneously. The distributional features of health utility data make such mapping approaches sub-optimal. The ISPOR Good Practice Guide recognises this and recommends the use of models that are less likely to suffer from bias [16]. More recently, a HTA report has demonstrated how recently developed bespoke methods have been repeatedly shown to perform well for this purpose [17]. Furthermore, Woodcock and Doble mapped QLQ-C30 to EQ-5D in cancer patients, some of whom had breast cancer, and compared linear models to response mapping, 2-part beta models and mixture models. They found that the 2-part beta models performed best overall, OLS estimated EQ-5D well when patients were in good health, but that mixture models performed better for patients in poor health [18].

## Methods

### Data

The data used in this study is from the trastuzumab emtansine versus treatment of physician’s choice for pretreated HER2-positive advanced breast cancer (TH3RESA) study. The TH3RESA study was a phase 3 open-label randomized controlled trial that randomized patients between 2011 and 2012. It includes data on 602 patients who were recruited from medical centres across 22 countries in Europe, North America, South America, and Asia-Pacific. Eligible patients were adults who had HER2-positive, recurrent or unresectable locally advanced breast cancer or metastatic breast cancer and had previously received taxane therapy in any setting or more than one HER2-directed regimens in an advanced setting; these included trastuzumab and lapatinib. Patients were randomly assigned in a ratio of 2:1 to trastuzumab emtansine (3.6 mg/kg intravenously every 21 days) or the physician’s choice of treatment [19]. Patients were enrolled if they had non-measurable or measurable disease defined by the Response Evaluation Criteria in Solid Tumors (RECIST) version 1.1. Patients completed the EORTC QLQ-C30 [20] version 3c [21] and the EQ-5D-3L questionnaire simultaneously, on the first day of each treatment cycle until disease progression (assessed by the investigator) or their treatment was discontinued. Multiple observations are recorded for each individual so we provide robust standard errors. UK value sets [22] are used throughout.

Observations with missing data on variables used in the mapping algorithms are removed from analysis. As this is trial data, we do not believe that missing data will influence results.

### Statistical analysis

The distributional characteristics of EQ-5D-3L are such that standard statistical models for mapping are inappropriate and tend to lead to biased estimates [7, 9, 13, 17]. A bespoke method for directly mapping to EQ-5D-3L utility scores based on an adjusted limited dependent variable mixture model (ALDVMM) approach has been validated for mapping in several different disease areas [7–9, 23, 24]. This method was applied in Stata v14, using the publicly available Stata command ‘aldvmm’ [25]. The command was developed specifically for estimation of the EQ-5D-3L. Here we use the UK tariff, but the command can also be used for any international tariff, the EQ-5D-5L and other health state utility measures. In brief, each component in the mixture is normally distributed and limited at full health [1] and below at the “pits” state, 33,333 (− 0.594). The command allows a gap in the distribution between full health and the next feasible value (in the UK case this is 0.883) corresponding to the EQ-5D-3L. Therefore, even a single component model is capable of reflecting a mass of observations at the upper full health limit, and does not produce values in the three non-feasible areas: above 1, below − 0.594 or between 0.883 and 1. Mixing more than one of these components adds flexibility to reflect other non-normal characteristics of the typical EQ-5D distribution such as multimodality.

We considered different numbers of components within the mixture models and chose the optimal number of components based on a number of different measures of fit (mean error, root mean squared error (RMSE), Akaike, Bayesian and Quasi information criteria (AIC, BIC and QIC, respectively)) and judgements about parsimony of the model. For the sake of comparison, we also estimated a random effects linear regression model and response mapping using seemingly unrelated ordered probit models. Response mapping is the term used for a two stage mapping approach. Instead of directly modelling the EQ-5D utility scores, response mapping first estimates the probability of responding at level 1, 2 or 3 on each of the five dimensions of health, independently, as a function of QLQ-C30 and other covariates. Stage two then calculates the expected utility scores based on the probabilities for each of the 243 EQ-5D-3L states.

We estimate seemingly unrelated ordered probit models which combine the estimation results of the five EQ-5D dimensions into a single parameter vector and simultaneous variance-covariance matrix. This is done using the *cmd* Stata command for estimating conditional mixed process models with multilevel random effects and coefficients.

The QLQ-C30 can be reported at different levels of aggregation. Different options therefore arise for the analysis. In its most granular form, each of the individual 30 questions can be entered as explanatory variables. However, clinical studies rarely report the results to each individual question, limiting the usefulness of any mapping to those situations where the economic analyst has access to the raw patient level data. Furthermore, there are clear correlations between many of the questions which provides a rationale for combining them into subscale scores. For example, the nausea and vomiting scale is formed from two questions [26]. We therefore undertook analyses that used the global health status scale, functional and symptom scales and items. In all cases, we rescaled these variables by dividing by 100. We also included age as a covariates, but not sex due to the small proportion of males in the sample (< 0.25%). In the case of the mixture models, the subscale scores are included as covariates in the individual components. Global health status and the square of global health status were included as covariates influencing the probability of component membership.

To compare models, we use a range of measures and visual techniques, as well theoretical appropriateness of the model to determine which the best model is. This is in accordance with the ISPOR good practice guide [16].

We report widely used measures of overall model fit such as mean absolute error (MAE) and root mean squared error (RMSE) as well as comparing the mean predicted and mean observed values. We also use Akaike, Bayesian and Quasi Information Criteria to compare models with the same dependent variables. In addition, we use visual measures of fit. This is because the measures reported above only provide a summary of overall fit. For use in economic evaluation, mapping models need to consider how different models fit, and how this may differ, across the entire health distribution. We will use cumulative distribution functions, showing the actual and predicted data to determine how closely each model fits the data across the distribution. We will also use the mean observed versus the mean expected values of EQ. 5D across different values of global health status allowing us to examine model performance across the spectrum of disease.

Next, we take the preferred models and using simulated data, compare the results using this model to the original data. This allows us to assess how well the chosen model will estimate any uncertainty in the model.

## Results

### Dataset

From a total of 3817 observations, 161 (4.2%) were dropped due to missing values for EQ-5D or QLQ-C30 scores, leaving a sample of 3201 observations. Table 1 reports summary statistics on patient characteristics at baseline and quality of life for all time points in the patients included in our sample. All but 9 of the 602 patients were female and mean age was 54 years. At baseline, most patients had stage II (31%) or stage III (37%) disease. The sample spanned the entire range of disease severity measured by EQ-5D (− 0.594 to 1), with 111 distinct health states of the 243 that can be described by EQ-5D.

**Table 1:** Sample descriptive statistics

	***n***	***Mean or %***	***Sd***	***Min***	***Max***
Baseline age (yrs)	602	53.6	10.5	27	89
EQ-5D	3766	0.715	0.263	−0.594	1
Global health status/QoL	3816	61.85	22.82	0	100
Physical Function	3817	76.74	21.98	0	100
Role Function	3817	70.84	29.47	0	100
Emotional Function	3817	74.67	22.99	0	100
Cognitive Function	3817	81.01	21.94	0	100
Social Function	3817	74.14	28.38	0	100
Fatigue	3817	35.67	25.65	0	100
Nausea / vomiting	3817	7.47	14.72	0	100
Pain	3817	27.32	27.49	0	100
Dyspnoea	3817	18.3	25.75	0	100
Insomnia	3817	27.05	28.85	0	100
Appetite loss	3817	16.77	26.39	0	100
Constipation	3817	19.53	27.56	0	100
Diarrhoea	3817	6.58	16.65	0	100
Financial problems	3817	21.47	31.37	0	100
***Sex - female***	598	99.34			
***Baseline Disease Stage***					
Stage 0	6	1.02			
Stage I	70	11.93			
Stage IIA	103	17.55			
Stage IIB	81	13.8			
Stage IIIA	111	18.91			
Stage IIIB	58	9.88			
Stage IIIC	41	6.98			
Stage IV	117	19.93			

**Fig. 1:**
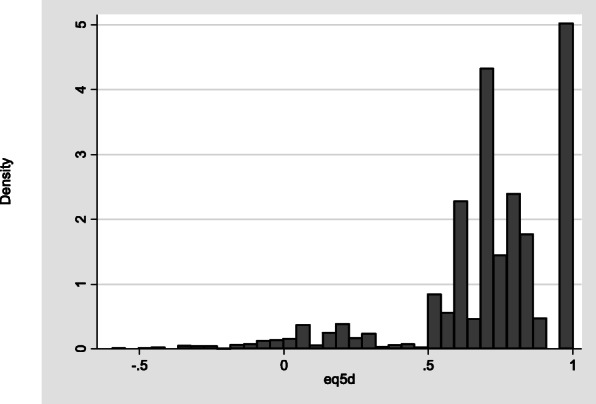
Histograms of EQ-5D-3L Utility Values

**Fig. 2:**
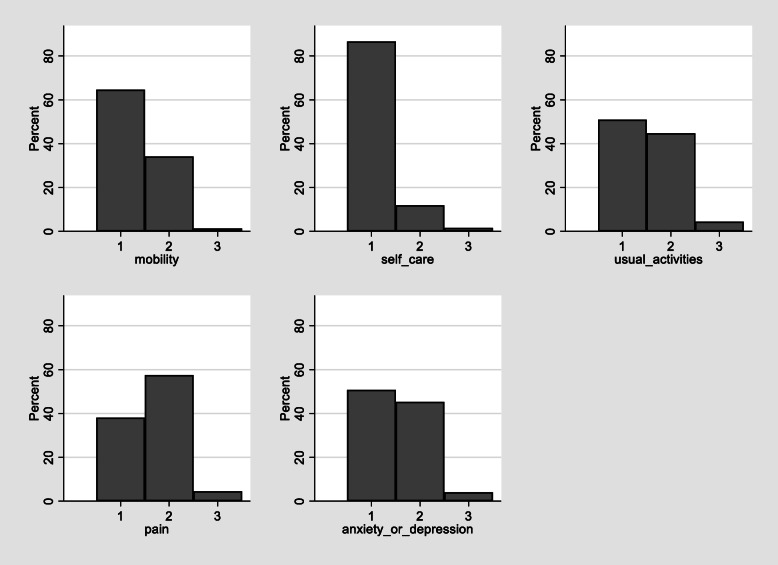
Distribution of EQ-5D responses by dimension

Figure 1 shows the data exhibit characteristics typical for EQ-5D. Mean EQ-5D was 0.715. Around 3% of observations or 101 observations were below zero and 861 (23%) were equal to one. The distribution (displayed in Fig. 1) is tri-modal and has a gap between the mass of observations at full health and the next feasible value (0.883). Figure 2 displays the distribution of responses to the five EQ-5D dimension questions. It shows that whilst all response categories are included in the sample, the number responding level “3”, the greatest level of impairment, are relatively low. Only 1% of respondents reported being at level 3 for mobility or self-care.

QLQ-C30 scores spanned their entire feasible 0–100 range for the global health status assessment and each of the functional and symptom scales as well as the single items. High scores for the global score and the functional scales represent higher levels of quality of life whereas high scores for the symptom scales and items correspond to high levels of symptomatology. “Fatigue” has a mean score of 36 and is the dimension with the highest level of problems.

### Model results

We found that a linear regression suggested that “role functioning” and “appetite loss” were insignificant in estimating EQ-5D. These were also non-significant, inter alia, in the mapping work of Crott and Briggs and were removed from the final analysis in their stepwise regression approach [3]. However, when using response mapping we found that these scores were statistically significant in estimating some of the domains of EQ-5D. We therefore kept these scores in our analysis.

We estimated ALDVMM models with different numbers of components and with different variables within components and as predictors of component probabilities. The preferred models included global health status, the functional scales, the symptom scores/items as well as age to predict each component. Each of these components has a probability which is a function of a set of independent variables. The probabilities of each component were determined by global health status and its squared term. We tried different combinations of variables predicting the component probabilities but found that including only global health and global health squared performed best. This is in line with previous research [17] showing that some measure of disease severity typically should be included as predictors of component membership.

In order to make the results more comparable, all explanatory variables included within components and component probabilities are included in the linear model and response mapping, we also include age squared in order to add further flexibility to the linear and response models. Table 2 shows summary fit statistics for ALDVMM models with 1 to 4 components, as well as for the response mapping approach estimated using seemingly unrelated ordered probit models and a linear model estimated using OLS.

**Table 2:** Comparisons of fit statistics

	MAE	RMSE	AIC^**a**^	BIC^**a**^	QIC^**a**^	Mean^**b**^	Absolute Difference
Linear regression	0.1203	0.1680	–	–	–	0.7103	0.0047
Response mapping	0.1191	0.1702	–	–	–	0.7057	0.0093
1 component ALDVMM	0.1196	0.1696	− 2302.11	− 2164.82	− 2289.01	0.7201	0.0051
2 component ALDVMM	0.1173	0.1684	− 2538.83	−2164.82	− 2515.96	0.7201	0.0051
3 component ALDVMM	0.1172	0.1677	− 2528.40	− 2154.39	− 2459.09	0.7171	0.0021
4 component ALDVMM	0.1173	0.1675	− 2673.51	− 2168.59	− 2629.49	0.7182	0.0032

*MAE* Mean Absolute Error, *RMSE* Root Mean Squared Error, *AIC* Akaike Information Criteria, *BIC* Bayesian Information Criteria

^a^Note that AIC, BIC and QIC cannot be compared between linear regression, response mapping and mixture models and so they are not included for the linear model or response mapping

^b^mean observed = 0.7150

The random effects linear regression model appears to fit the data reasonably well based on summary fit measures. It has a low root mean squared error (RMSE) and the predicted mean is close to that of the observed data. The response mapping is less accurate at estimating the mean EQ-5D-3L and has a larger RMSE than all other models, however, the mean absolute error (MAE) is marginally lower for the response mapping than for the linear model. In general, the ALDVMMs outperform both the linear and response mapping when looking at the MAE, RMSE and mean prediction, particularly when looking at the 3- and 4- component models. Note that AIC, BIC and QIC cannot be compared between the linear regression, response mapping and the mixture models.

Of the four ALDVMMs, the 4-component model is preferred. It has the lowest RMSE, the estimated mean is close to that of the observed data and it has the lowest AIC, BIC and QIC values. Model preference was determined on the summary fit statistics given in Table 2 as well as looking visually at the distribution of EQ-5D and how the models with different numbers of components fit the observed data.

**Fig. 3:**
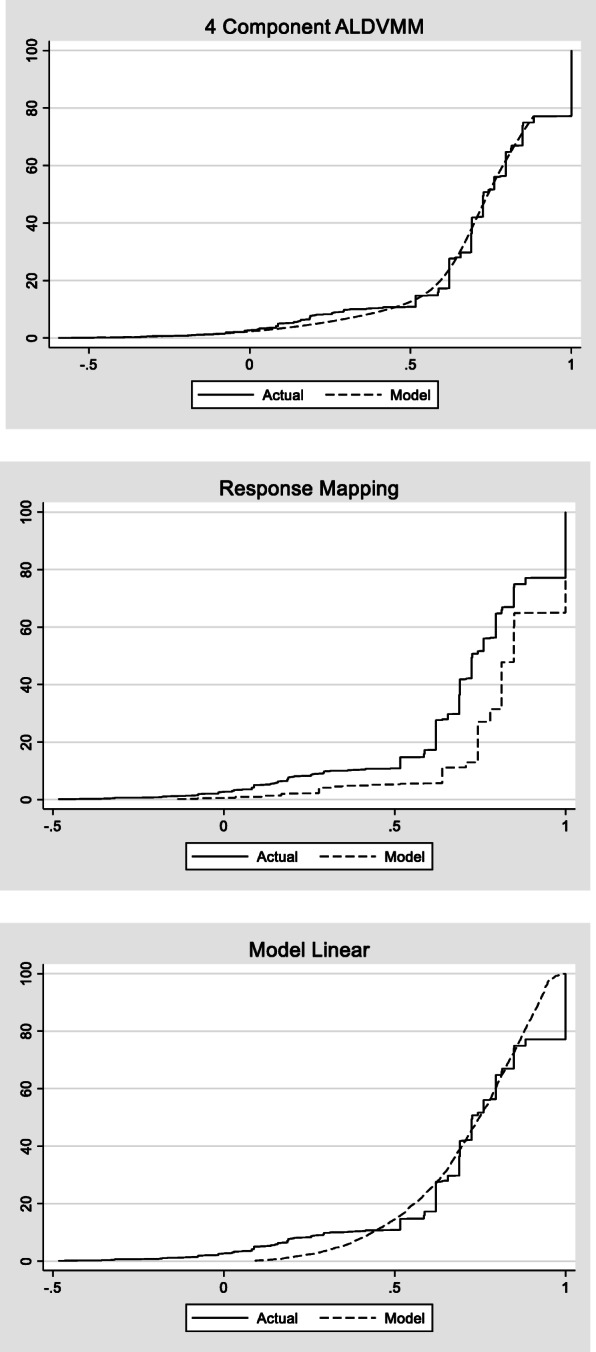
Cumulative distribution function for 4 component ALDVMM, random effects linear model and response mapping using ordered probit models

In order to further compare the results of our models, we examined model performance across the spectrum of disease. This is because the summary fit statistics given in Table 2 do not tell us how the models fit at different parts of the distribution, they only give an average fit. With so many explanatory variables the most informative manner for displaying this is via the cumulative distribution function (CDF). The CDF for the 4-component ALDVMM, the response mapping and the linear model are presented in Fig. 3. The 4-component ALDVMM fits the observed data very closely across the spectrum of severity. Most notably, the ALDVMM exhibits little bias regardless of the health of the patient. There is a relatively large disparity in the observed data and predictions using response mapping across the distribution, potentially due to the small values that are observed in the most severe response for mobility and self-care (see Fig. 2). Although the linear model appears to predict the data better than the response mapping at certain parts of the distribution, it exhibits an under estimation of EQ-5D in those with good health and an overestimation in those in worse health as is generally the case when using linear models.

**Fig. 4:**
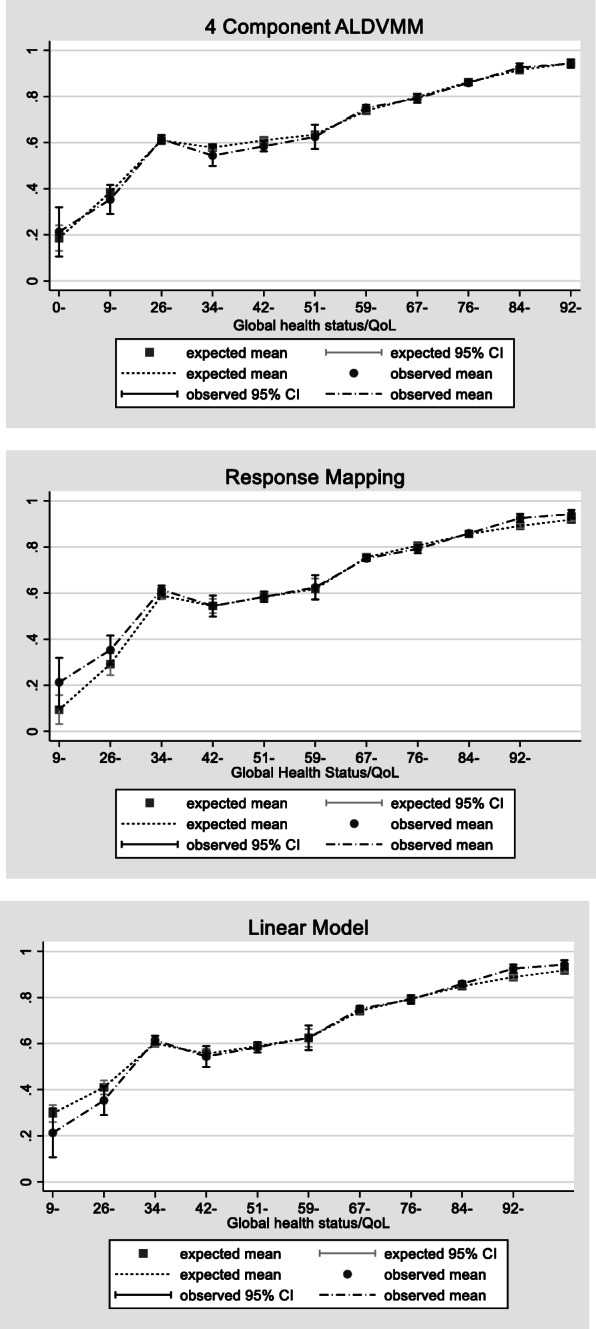
Mean expected vs Mean observed values for 4 component ALDVMM, random effects linear model and response mapping using ordered probit models

Figure 4 shows the mean observed and mean expected values of the global health measure of the QLQ-C30 in the 4-component ALDVMM, response mapping and linear regression. Again, the mixture model outperforms the linear model and the response mapping, showing very little difference between the observed and predicted values. Although both the response mapping and the linear model predict well in the centre of the global health score distribution, they both fail to predict well at either end of this distribution. It is worth noting that, in line with previous findings [7, 13], the linear regression shows the usual underestimation for those in good health and overestimation for those in poor health. Predicting well across the entire distribution is important if results are to be used in cost-effectiveness analyses in order to give unbiased and reliable conclusions.

Together, Table 2, Figs. 3 and 4 suggest that although the linear and response mapping models can predict mean values relatively well in this example, they can struggle to estimate well across all parts of the distribution, particularly for those in very good or very poor health. It is this aspect of fit that matters most when using a mapping function in an applied cost effectiveness analysis.

**Fig. 5:**
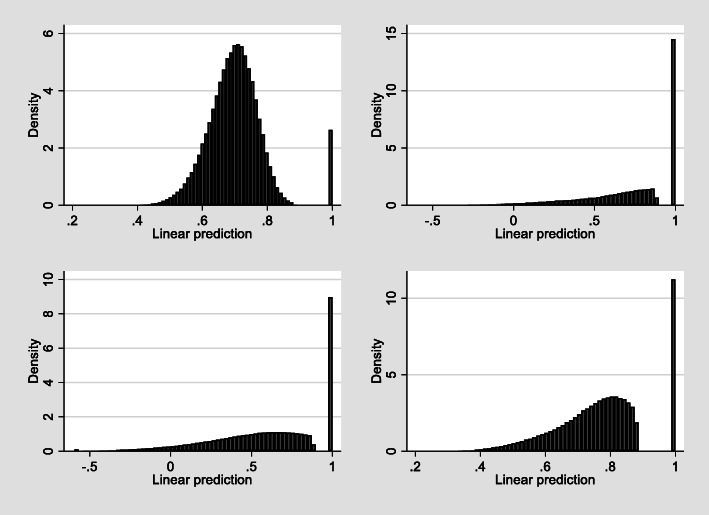
Distribution for each component of the preferred 4 component mixture model

In order to illustrate the predictive accuracy in terms of uncertainty of the preferred ALDVMM, we simulated 1000 data points from the 4 component ALDVMM model for each of the observations. Figure 5 shows the distribution of those simulations by component. The plot illustrates the gap in feasible values below full health, in all components.

There is a 0.2213 probability of observations being in component 1, which has a mean EQ-5D of 0.6720 (sd 0.070). Observations are most likely to be assigned to component 2 which has a mean EQ-5D of 0.8238 and standard deviation 0.242 with a probability of 0.3213. This component generates a substantial mass point at 1, full health. Observations are least likely to be in component 3 with a probability of 0.1686. Component 3 has a mean EQ-5D of 0.4740 and a standard deviation of 0.371. Both component 2 and 3 contribute to the entire range of EQ-5D. Component 4 has mean EQ-5D of 0.7781 and standard deviation 0.126 with observations having a 0.2888 probability of being assigned to it. Overall, the simulated data closely aligned to the original data. 22.15% of the simulated data were equal to 1 compared to 21.93% in the comparable original data. The estimated mean EQ-5D was 0.7180 compared to the observed mean of 0.7150. The variance was 0.0628 in the simulated data versus 0.0693 in the original data, and the skewness was − 1.502 in the simulated data and − 1.522 in the observed data. This use of simulated data compared with observed data shows that mapping can appropriately reflect uncertainty in utility responses well.

The mapping from QLQ-C30 to EQ-5D-3L using the 4 component ALDVMM estimated in this study can be implemented using Excel or Stata. An excel calculator and a Stata .stir file and corresponding .do file can be found in the supplementary materials. These files make it easy for the user to calculate the EQ-5D-3L from any combination of QLQ-C30 scores.

## Discussion

This paper provides estimates for the prediction of EQ-5D-3L as a function of QLQ-C30 scale scores and symptoms for patients with breast cancer, based on a large trial dataset comprising over 3800 observations. The estimates resulting from this study allow analysts to take results of clinical studies that did not collect EQ-5D to derive EQ-5D-3L utilities based on the QLQ-C30. Our results add to a growing literature demonstrating that many of the problems associated with the application of standard statistical methods, widely used in mapping, are largely eliminated by using the bespoke mixture model approach. This is further outlined by comparisons with Crott and Briggs who used OLS regressions to map from QLQ-C30 to EQ-5D-3L; Fig. 2 of their paper shows how using OLS in this context underestimated EQ-5D in healthy patients and overestimates it in those in poor health. Similarly, Young et al. [15], estimated EQ-5D from QLQ-C30 using a variety of model types (including linear regression, tobit, two-part models, splining and response mapping using multinomial logistic regression), in a dataset derived by combining separate studies of patients with breast cancer (*n* = 100), lung cancer (*n* = 99) and multiple myeloma (*n* = 572). In their study, all models exhibited substantial over-prediction of EQ-5D for those in poorer health, and this tendency extended over a wide range of EQ-5D. For values of over approximately 0.6 all models under-predicted EQ-5D. In general, models mapping between QLQ-C30 and EQ-5D have not performed well in different cancer types [3, 15, 27]. Methods that do not take into account the non-standard distributional characteristics of EQ-5D discussed previously have a tendency to result in bias. These biases matter because they result in new health technologies appearing less cost effective than they might truly be. The use of mixture models used in our study appears to overcome these problems. The results in this study are similar to those found in other applications including asthma [9], rheumatoid arthritis [8, 13], osteoarthritis [24], ankylosing spondylitis [7] and traumatic brain injury [28], in that they suggest mixture models are better equipped at predicting EQ-5D than other regression models.

These results contrast with those of Woodcock and Doble [18], in that we find that ALDVMM outperforms a linear model estimated using OLS in both patients with very good and very poor health.

Given the degree of variability in preference data of this type, the preferred 4-component model has an excellent predicted fit across the utility distribution. The unusual characteristics of the EQ-5D distribution are closely mirrored by the mixture model. In particular, it is worth noting that whilst it is often claimed that mapping substantially underestimates the uncertainty in utility responses, this study shows that this is not the case. By comparing simulated data to observed data we demonstrate that the results of this study produce good estimates of uncertainty. This is an important finding because if mapping algorithms are to be used in economic analysis, it is important that uncertainty as well as mean predictions can be estimated accurately.

The improvement in accuracy that the 4-component ALDVMM provides compared to the response mapping and linear prediction is both significant and important. Take Fig. 4, for example. The linear model shows a difference in predicted EQ-5D of 0.62 if patients were to move from the worst (0.30) to the best (0.92) health state described by QLQ-C30. The value of this same change using the estimates from the ALDVMM model is 0.78. The ALDVMM predicts poor health to yield a lower EQ. 5D score (0.16) and a higher good health score (0.95). This is not a trivial difference but a 26.4% increase from using a more suitable statistical model. The use of results from the linear model in an economic evaluation would risk undervaluing the benefits of an effective health technology.

The use of the ALDVMM model ensures that whether the mapping is used to populate health states for a decision model (such as pre and post progression) or to predict data for individual patients within a clinical study, its outputs will be coherent and consistent with the underlying EQ-5D instrument. In addition, values cannot be predicted outside the feasible zones as they can for other regression models.

## Conclusion

Utility mapping, from cancer specific scores such as QLQ-C30 to PBMs such as the EQ-5D-3L, underpin economic evaluations for decision making bodies. Many mapping methods which are commonly used are inappropriate and can undervalue the benefits of new treatments of cancer patients, making them seem less cost-effective than they are and therefore jeopardizing patient access to newly available treatments. This study provides a robust mapping method and corresponding algorithm (in the form of excel file or Stata file) which can allow benefits to patients to be appropriately assessed.

## Supplementary Information

**Additional file 1.** Excel Calculator.

## Data Availability

Data may be available from the authors upon reasonable request and only with permission of F. Hoffman La Roche.

## Abbreviations

ALDVMM: Adjusted limited dependent variable mixture model
AIC: Akaike information criteria
BIC: Bayesian information criteria
CDF: Cumulative distribution function
EORTC: European organisation for research and treatment of cancer
HER2: Human epidermal growth factor receptor 2
HTA: Health technology assessment
OLS: Ordinary least squares
PBM: Preference based measures
QALY: Quality adjusted life year
QIC: Quasi information criteria
QLQ: Quality of life questionnaire
RECIST: Response evaluation criteria in solid tumors
RMSE: Root mean squared error
TH3RESA: Trastuzumab emtansine versus treatment of physician’s choice for pretreated HER2-positive advanced breast cancer trial
