# Correction to: Mapping the EORTC QLQ-C30 to EQ-5D-3L in patients with breast cancer

**DOI:** 10.1186/s12885-022-09226-8

**Published:** 2022-01-27

**Authors:** Laura A. Gray, Monica Hernandez Alava, Allan J. Wailoo

**Affiliations:** Health Economics and Decision Science, School of Health and Related Research, University of Sheffield, Sheffield, UK

**Correction to: BMC Cancer 21, 1237 (2021)**

**https://doi.org/10.1186/s12885-021-08964-5**

Following publication of the original article [1], the authors reported an omission in the additional files. Additional file 2 and 3 were missing. The two file have been included in this correction article:

## Supplementary Information

**Additional file 2.** Out of Sample Predictions_QLQ-C30.do.

**Additional file 3.** QLQ-C30_4LC.ster.
